# Combined Silicon-Phosphorus Fertilization Affects the Biomass and Phytolith Stock of Rice Plants

**DOI:** 10.3389/fpls.2020.00067

**Published:** 2020-02-18

**Authors:** Zimin Li, Fengshan Guo, Jean-Thomas Cornelis, Zhaoliang Song, Xudong Wang, Bruno Delvaux

**Affiliations:** ^1^Soil Science, Earth and Life Institute, Université catholique de Louvain (UCLouvain), Louvain-la-Neuve, Belgium; ^2^School of Environment and Resources, Zhejiang Agricultural and Forestry University, Lin'an, China; ^3^BIOSE Department, Gembloux Agro-Bio Tech, University of Liege, Gembloux, Belgium; ^4^Institute of the Surface-Earth System Science, Tianjin University, Tianjin, China

**Keywords:** phytolith, crop yield, silicon-phosphorus fertilization, rice, silicon cycle

## Abstract

Phytoliths are silica bodies formed in living plant tissues. Once deposited in soils through plant debris, they can readily dissolve and then increase the fluxes of silicon (Si) toward plants and/or watersheds. These fluxes enhance Si ecological services in agricultural and marine ecosystems through their impact on plant health and carbon fixation by diatoms, respectively. Fertilization increases crop biomass through the supply of plant nutrients, and thus may enhance Si accumulation in plant biomass. Si and phosphorus (P) fertilization enhance rice crop biomass, but their combined impact on Si accumulation in plants is poorly known. Here, we study the impact of combined Si-P fertilization on the production of phytoliths in rice plants. The combination of the respective supplies of 0.52 g Si kg^–1^ and 0.20 g P kg^−1^ generated the largest increase in plant shoot biomass (leaf, flag leaf, stem, and sheath), resulting in a 1.3-fold increase compared the control group. Applying combined Si-P fertilizer did not affect the content of organic carbon (OC) in phytoliths. However, it increased plant available Si in soil, plant phytolith content and its total stock (mg phytolith pot^−1^) in dry plant matter, leading to the increase of the total amount of OC within plants. In addition, P supply increased rice biomass and grain yield. Through these positive effects, combined Si-P fertilization may thus address agronomic (e.g., sustainable ecosystem development) and environmental (e.g., climate change) issues through the increase in crop yield and phytolith production as well as the promotion of Si ecological services and OC accumulation within phytoliths.

## Introduction

Amorphous biogenic silica (SiO_2_·nH_2_O) can accumulate in living plant tissues during their growth and development (Conley, 2002; Piperno, 2006). These silica bodies, named phytoliths, are released into the soil after the decomposition of litter and plant residues (Smithson, 1956; Alexandre et al., 1997; Fraysse et al., 2006). Depending on their chemical composition and structure, phytoliths can accumulate in soils and sediments over centuries or millennia, or dissolve and then contribute to the pool of aqueous monosilicic acid (dissolved silicon: DSi), which is available for plant root uptake (Bartoli, 1985; Meunier et al., 1999; Fraysse et al., 2009; Struyf et al., 2010; Cornelis and Delvaux, 2016). The elemental composition of phytoliths is influenced by plant species and phytolith morphology (Bartoli and Wilding, 1980; Li et al., 2014; Nguyen et al., 2014). Organic carbon (OC) (0.2–6%) can be associated with phytoliths (Bartoli, 1985; Parr and Sullivan, 2005; Parr et al., 2010; Zuo and Lü, 2011; Li et al., 2013c; Alexandre et al., 2015). In particular, the occlusion of organic carbon (OC) within phytolith (PhytOC), which is formed in plant tissues, has been proposed as a mechanism which traps the photosynthesized molecules within silica bodies (Parr and Sullivan, 2005; Santos et al., 2012; Alexandre et al., 2015; Reyerson et al., 2016). The occurrence of PhytOC has been reported in various studies (Parr and Sullivan, 2005; Parr et al., 2010; Parr and Sullivan, 2011; Song et al., 2012; Song et al., 2013; Li et al., 2013a; Li et al., 2013b; Huang et al., 2014; Song et al., 2015; Guo et al., 2015; Sun et al., 2016; Pan et al., 2017; Qi et al., 2017; Li et al., 2018a). However, the biological processes leading to the occurrence of PhytOC has not been demonstrated. Therefore, OC content in phytoliths varies depending on the extraction procedure (Parr and Sullivan, 2014; Santos and Alexandre, 2017; Song et al., 2016). These variations led to a debate on the scale of OC occlusion within phytoliths, and on the significance of the PhytOC sink for the global C cycle and climate change mitigation (Parr and Sullivan, 2005; Song et al., 2012; Hodson, 2016; Reyerson et al., 2016; Lorenz and Lal, 2018; Crifò and Strömberg, 2019; Ramesh et al., 2019; Song et al., 2016; Santos and Alexandre, 2017). In addition, OC associated with phytoliths might have a non-photosynthetic origin attributed to the uptake of organic molecules from soil (Santos et al., 2012; Alexandre et al., 2015; Reyerson et al., 2016), which could lead to erroneous C dating using phytoliths (Hodson, 2016). Therefore, the accurate determination of the phytolith OC content must not only completely eliminate external OC, but also keep the phytolith structure intact and the oxidation of C in the phytolith to a minimum (Parr and Sullivan, 2014). Overoxidation may significantly underestimate phytolith C sequestration and should be avoided (Parr and Sullivan, 2014).

In any case, whether phytoliths sequester OC or not, the increase in silicon (Si) uptake undoubtedly enhances plant biomass, Si and phytolith content in plants [a.o. (Li et al., 2018b; Li et al., 2019)]. The amount of OC that could be associated with phytoliths would depend on plant Si accumulation and thus phytolith content (Li et al., 2013c); therefore, suggesting that regulating Si supply might increase phytolith-associated OC in croplands. In this respect, the combination of Si and phosphorus (P) fertilization may enhance the contents of plant phytolith and OC associated within phytoliths.

This study is how co-fertilization combining Si and phosphorus (P) can affect Si availability and plant uptake, as well as phytolith formation in rice. Si uptake improves the growth of Si-accumulator cereals such as rice (Savant et al., 1997; Ma et al., 2001; Ma et al., 2006; Liang et al., 2015). Si fertilization can enhance rice resistance to biotic and abiotic stresses (e.g., pests, water and heat stress, disease, etc.) (Liang et al., 2007; Cooke et al., 2016; Cooke and Leishman, 2016; Coskun et al., 2019), and thus promote rice crop yields and Si accumulation (Savant et al., 1997; Ma et al., 2001; Keller et al., 2012). However, P fertilization also plays an important role in improving yields and promoting plant precocity (George et al., 2001; Lambers et al., 2006; Hammond and White, 2008). In paddy soils, Si and P fertilization could alleviate P deficiency, increase P uptake by plants (Ma and Takahashi, 1990; Liang et al., 2007; Hu et al., 2018), and enhance plant available Si in soil, hence improving crop yields (Song et al., 2014; Klotzbücher et al., 2015; Carey and Fulweiler, 2016; Li et al., 2019). Furthermore, plant available Si content in soil may increase after P supply. Besides, Si supply can increase P bioavailability in soil through the competition between silicate and phosphate for sorption on Al and Fe oxide surfaces that bear positive charges (Parfitt, 1989; Su and Puls, 2003). Combined Si-P fertilization may thus substantially influence Si and P biocycling in the soil-plant system, as well as plant phytolith and chemical composition.

Through a pot experiment in controlled conditions, we aim to address three interconnected questions: 1) does Si-P fertilization increase rice biomass? 2) does increased biomass promote plant phytolith formation? and 3) does combined Si-P supply impact the amount of OC associated within phytoliths?

## Materials and Methods

The pot experiment was carried out at Zhejiang Agricultural and Forestry University, Lin'an, Zhejiang Province, Eastern China (29°56'–30°27'N, 118°51'–119°52'E). This region is characterized by a mid-subtropical monsoon climate with a mean annual precipitation of 1,500 mm, a mean annual temperature of 15.8°C, 237 frost-free days, and an annual 1,939 h of sunshine.

### Pot Experiment Design and Management

The soil used was a Cambisol, according to the World Reference Base (WRB) key (IUSS, 2014), sampled from the agricultural station at Zhejiang Agricultural and Forestry University. The soil was air-dried, sieved at 2 mm, and mixed with Si-P fertilizers. The soil physico-chemical properties were as follows (Lu, 2000): pH_water_ = 5.34 ± 0.02, soil organic matter = 30.26 ± 4.28 g kg^−1^, available Si = 155.59 ± 22.73 mg kg^−1^, available P = 113.87 ± 1.35 mg kg^−1^, available K = 10.33 ± 1.11 mg kg^−1^ and available N = 87.15 ± 2.47 mg kg^−1^ (Guo et al., 2015). The analytical methods were described by Lu (2000). Here, plant available Si was assessed using extracts of NaOAc and acetic acid. Jiayu 253 was selected as the experimental rice (*Oryza sativa*) cultivar because of its high yield and wide distribution in Zhejiang province.

The experiment was carried out using three fertilization levels, zero (0), medium (m), and high (h), for Si (Si_0_: 0, Si_m_: 0.26, Si_h_: 0.52 g SiO_2_ kg^–1^) using Na_2_SiO_3_, and P (P_0_: 0, P_m_: 0.2, P_h_: 0.4 g kg^–1^) using P_2_O_5_. Nine treatments (Si_0_P_0_, Si_0_P_m_, Si_0_P_h_, Si_m_P_0_, Si_m_P_m_, Si_m_P_h_, Si_h_P_0_, Si_h_P_m_, and Si_h_P_h_) and five replicates per treatment were set up (**Table 1**). N and K fertilizers were applied in all treatments as, respectively, urea ammonium nitrate (0.20 g N kg^−1^), and KCl (0.25 g K kg^−1^). All fertilizers were added to soil before planting rice. Soil pH value and available Si and P contents under different levels of Si and P supply were determined by Sun et al. (2015), as presented in **Table 2**. Each pot (0.24 m diameter, 0.28 m height) contained 8.5 kg air-dried soil and was regularly irrigated using tap water (Si: 0.36 μg L^–1^) at the same level until rice grain harvesting. After a first irrigation of 500 ml, 1,000 ml of water were supplied per pot during the whole growing period, once every 2 days. Crop harvesting was done 4 months after planting. The rice plant parts were sampled separately: sheath, leaf, flag leaf, and stem. Plant samples were thoroughly washed with deionized water, and then oven dried at 75°C until a constant weight was attained, as equal to dry shoot biomass. Rice grains, including rice husk, were also dried at 75°C and weighed.

**Table 1:** The pot experimental design, as designed following silicon (Si) and phosphorus (P) levels. Different lowercase letters indicate significant differences among all treatments [least significant difference (LSD) test; p < 0.05, n = 5].

Number	Treatments	SiO_2_ fertilizer quantity (g kg^−1^)	Si fertilizer levels	P_2_O_5_ fertilizer quantity (g kg^−1^)	Phosphoric fertilizer levels
1	Si_0_P_0_	0.00	Low	0.0	Low
2	Si_0_P_m_	0.00	Low	0.2	Medium
3	Si_0_P_h_	0.00	Low	0.4	High
4	Si_m_P_0_	0.26	Medium	0.0	Low
5	Si_m_P_m_	0.26	Medium	0.2	Medium
6	Si_m_P_h_	0.26	Medium	0.4	High
7	Si_h_P_0_	0.52	High	0.0	Low
8	Si_h_P_m_	0.52	High	0.2	Medium
9	Si_h_P_h_	0.52	High	0.4	High

**Table 2:** Soil pH value and available silicon (Si) and phosphorus (P) contents under different levels of Si and P supply^*^.

Treatments	pH	Available P	Available Si	Available N	Available K
		mg kg^−1^			
Si_0_P_0_	5.47d	10.25d	102.45cd	103.37c	10.25d
Si_0_P_m_	5.50d	14.58c	112.23c	104.19bc	14.58c
Si_0_P_h_	5.59cd	17.84b	110.23c	107.40bc	17.84b
Si_m_P_0_	5.67c	14.23c	123.48bc	98.81c	14.23c
Si_m_P_m_	5.71c	17.22b	132.79b	117.87b	17.22b
Si_m_P_h_	5. 77c	19.27a	133.28b	127.00a	19.27a
Si_h_P_0_	6.49a	14.29c	142.14ab	98.17c	14.29c
Si_h_P_m_	6.22b	18.06ab	153.83a	98.75c	18.06ab
Si_h_P_h_	6.38ab	20.33a	155.22a	111.13b	20.33a

*The data were collected from Sun et al., 2015.

### Plant Chemical Analysis

Dried plant samples were cut into small pieces by stainless steel scissors for the analysis of Si and phytolith content. Plant samples were fused with Li-metaborate at 950°C and dissolved in nitric acid (HNO_3_ 4%), prior to molybdenum blues colorimetry to determine Si content (Lu, 2000).

**Figure 1:**
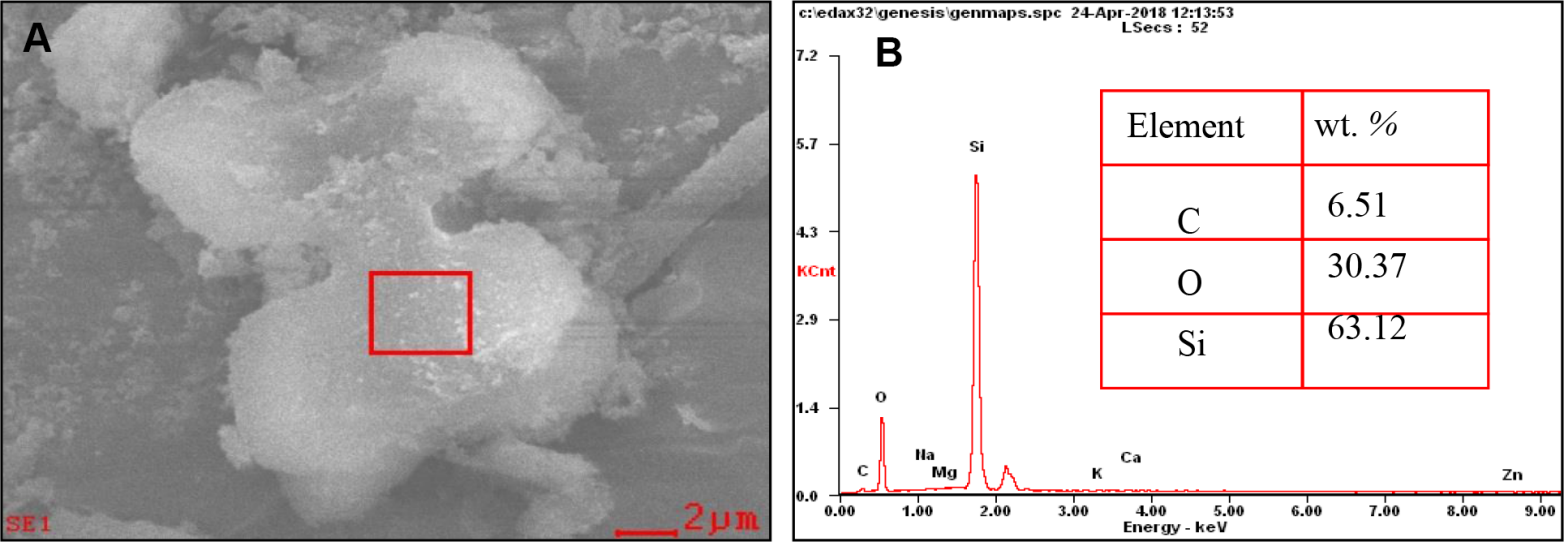
**(A)** Scanning electron microscope (SEM) image of rice leaf phytolith. **(B)** Semi-quantitative element concentration (wt. %, n = 5) measured by SEM-energy-dispersive spectroscopy (EDS) of the selected area.

Microwave digestion in combination with Walkley–Black digestion was used to isolate the phytoliths from plant material (Walkley and Black, 1934; Parr et al., 2001), in order to remove extraneous organic materials thoroughly (Li et al., 2013c). We first checked the presence of phytoliths by optical microscopy to ensure that all extraneous organic materials had been removed (Li et al., 2013c). Then, we further assess the purity of phytolith extract using the scanning electron microscope (SEM) images and energy-dispersive spectroscopy (EDS) (**Figure 1**). The phytoliths were then oven dried at 75°C for 24 h, cooled and weighed. Phytolith particles were dissolved in HF 1 M at 45°C during 100 min, so that associated OC could be released in the acidic solution (Li et al., 2013c). Associated OC content was determined using the potassium dichromate procedure and the soil standard reference GBW07405, ensuring a relative precision below 5% (Li et al., 2013c). Using plant dry matter, OC and phytolith contents, we computed OC_phyt_ and OC_pdm_, as the OC contents per mass unit of, respectively, phytolith and plant dry matter.

### Data Treatment

Phytolith stock (mg pot^−1^) = phytolith content (mg g^−1^) × biomass of dry plant tissue (g pot^−1^) where phytolith stock is used to refer to the mass of phytoliths per pot (mg pot^−1^); phytolith content is used to refer to the mass of phytoliths per gram of dry plant tissue (mg g^−1^); biomass of dry plant tissue is used to refer to the mass of dry plant tissue per pot (g pot^−1^).

A two-way analysis of variance of was performed to assess the effects of combined Si-P fertilization levels using SPSS (24.0). Fisher's least significant difference (LSD) test was used to compare the average values of the contents of SiO_2_, phytolith, OC_phyt_, OC_pdm_ in the different plant parts (leaf, flag leaf, sheath, and stem) (at *P* < 0.05 level, n = 5). Origin 8.0 software was used to plot the figures.

## Results

### Rice Shoot Biomass and Grain Yield

The rice shoot biomass (g pot^−1^) significantly varied from 168 in Si_0_P_0_ to 213 in Si_m_P_m_ or Si_m_P_h_ (**Table 3**). Among the Si_0_ treatments, there was a significant increase in shoot biomass between Si_0_P_0_ and Si_0_P_h_ whereas Si_0_P_m_ was intermediate between and not significantly different from the other two treatment levels (**Table 3**). At the given level P_m =_ 0.2 _g_ kg^−1^, increasing Si application rate from Si_0_ to Si_m_ increased the leaf and shoot biomass (**Table 3**). At the same P_m_ level, rice grain yield increased from Si_0_ to Si_m_ and from Si_0_ to Si_h_ (**Table 3**).

**Table 3:** Effect of silicon-phosphorus (Si-P) levels on biomass in different plant parts and rice dry shoot.

Treatments	Leaf	Flag leaf	Stem and sheath	Grains^*^	Rice dry shoot
		g pot^−1^			
Si_0_P_0_	12.40c	5.65bc	55.62b	102.20c	175.88b
Si_0_P_m_	15.46a	6.20ab	53.64b	117.05bc	192.35ab
Si_0_P_h_	15.76a	5.53b	51.53b	130.39a	203.21a
Si_m_P_0_	9.98d	4.81c	42.80c	110.55c	171.70ab
Si_m_P_m_	15.22a	5.30bc	55.81b	136.42a	212.92a
Si_m_P_h_	13.34b	5.60bc	49.36bc	141.69a	210.99a
Si_h_P_0_	12.39c	4.72c	47.91bc	117.60bc	182.61ab
Si_h_P_m_	15.59a	7.08a	59.55ab	129.51ab	210.07ab
Si_h_P_h_	15.15a	5.50bc	62.28a	114.23bc	197.15ab

The data of rice organ (leaf, flag leaf, stem and sheath) collected from Sun et al., 2015;

Different lowercase letters represent significant differences of rice shoot biomass (Duncan's multiple range test; at p < 0.05 level, n = 5).

*Grains including rice husk.

### Content and Stock of Phytoliths Formed in Rice Plants

**Figure 2:**
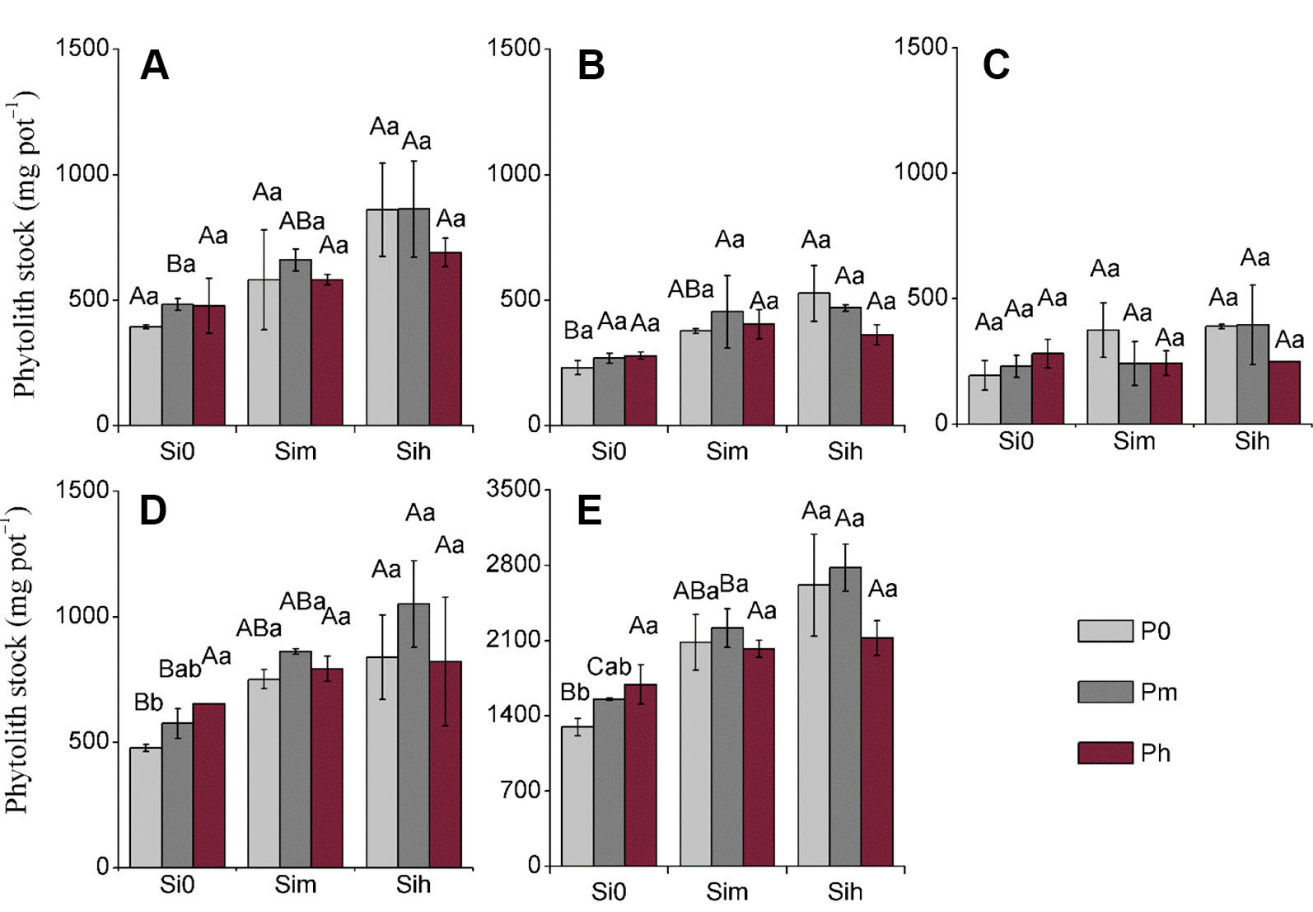
Phytolith stock (mg pot^−1^) at a two-way analysis of variance of silicon-phosphorus (Si-P) levels in the different plant parts. **(A)** Leaf; **(B)** flag leaf; **(C)**: stem; **(D)**: sheath; **(E)** rice shoot. Error bars represent the standard deviations of the means. Different lowercase letters indicate significant differences among the treatments in different P treatments and rice plant parts at a given Si level, respectively [least significant difference (LSD) test; *p ≤* 0.05, n = 5]. Different uppercase letters indicate significant differences among the treatments in different Si treatments and rice plant parts at a given P level, respectively (LSD test; *p* ≤ 0.05, n = 5).

Considering all plant parts, phytolith content significantly varied (*p* < 0.05) from 4.73 to 59.12 mg g^–1^ (**Tables 4**–**6**). At all given levels of Si_0_, Si_m_, and Si_h_, the increase in P application rate did not significantly increase phytolith content regardless of plant part, while this effect was not true for sheath with a significant increase from Si_0_P_0_ to Si_0_P_m_ (**Table 5**). Yet at given levels P_0_, P_m_, and P_h_, the increase in Si application rate significantly increased phytolith content in all plant parts (**Table 5**). Phytolith content in leaves was the highest, and varied from 28.36 to 59.12 mg g^–1^, with an average of 39.82 mg g^–1^ (**Table 4**). As compared to the other plant parts, stem phytolith content was the lowest, with an average value of 7.11 mg g^–1^. Considering all plant parts, the stock of phytolith formed during the experimental period varied significantly from 152.6 to 1,002.7 mg pot^−1^ (**Figure 2**). Si-P fertilization increased the stock of phytoliths formed in all plant parts, including plant shoot biomass (**Figure 2**). At all given levels of Si_0_, Si_m_, and Si_h_, the increase in P application rate did not significantly increase phytolith stock regardless of plant part, including in plant shoot biomass (**Figure 2**; **Table 5**). Yet at given levels P_0_, P_m_, and P_h_, the increase in Si application rate significantly increased phytolith stock in all plant parts, including plant shoot biomass. The mean phytolith stock was the highest in sheath (758.3 mg pot^−1^), followed by leaf (621.0 mg pot^–1^), flag leaf (374.3 mg pot^–1^), and stem (average 289.1 mg pot^–1^). Considering shoot biomass and including rice grains, the stock of phytolith significantly varied from 1,296.6 to 2,778.6 mg pot^−1^, the latter and maximal value being measured at Si_h_P_m_ level (**Figure 2**).

**Table 4:** Contents of SiO_2_, phytolith, organic carbon (OC) associated with phytolith as expressed per unit mass of phytolith (OC_phyt_) and of plant dry matter (OC_pdm_) in different plant parts (leaf, flag leaf, sheath, and stem).

Rice organs	Treatment		SiO_2_ content in plant parts	Phytolith content	OC_phyt_	OC_pdm_
	Si treatment	P treatment	(mg g^–1^)			
**Leaf**	Si_0_	P_0_	35.28 ± 5.25Ba	28.35 ± 3.69Ca	14.48 ± 1.62Aa	0.41 ± 0.10Ba
		P_m_	34.47 ± 4.21Ca	31.52 ± 0.70Ba	14.50 ± 0.41Aa	0.46 ± 0.02Ba
		P_h_	33.46 ± 5.88Ba	29.39 ± 3.03Ba	15.16 ± 1.93Aa	0.45 ± 0.10Aa
	Si_m_	P_0_	42.35 ± 5.76Ba	42.37 ± 6.98Ba	15.48 ± 2.86Aa	0.67 ± 0.23ABa
		P_m_	42.92 ± 0.62Ba	38.61 ± 4.07Ba	13.77 ± 0.53Aa	0.53 ± 0.08ABa
		P_h_	44.74 ± 3.95Aa	34.80 ± 5.08Ba	13.73 ± 2.47Aa	0.49 ± 0.16Aa
	Si_h_	P_0_	67.05 ± 2.84Aa	59.12 ± 1.39Aa	14.71 ± 3.51Aa	0.87 ± 0.23Aa
		P_m_	54.31 ± 5.03Ab	50.56 ± 4.86Ab	13.11 ± 1.67Aa	0.67 ± 0.15Aab
		P_h_	49.55 ± 0.45Ab	43.55 ± 0.30Ac	11.16 ± 0.92Aa	0.49 ± 0.04Ab
		**Mean ± s.d**.	**44.90 ± 10.86A**	**39.82 ± 10.33A**	**14.01 ± 1.30A**	**0.56 ± 0.15A**
**Sheath**	Si_0_	P_0_	21.85 ± 0.95Ca	19.47 ± 1.49Bb	16.74 ± 4.08Aa	0.33 ± 0.10Aa
		P_m_	23.99 ± 0.09Ba	21.73 ± 3.79Bb	18.04 ± 0.10Aa	0.39 ± 0.07Aa
		P_h_	24.31 ± 0.43Ca	23.44 ± 0.49Ca	18.17 ± 1.96Aa	0.43 ± 0.05Aa
	Si_m_	P_0_	35.85 ± 1.87Ba	32.00 ± 1.84Aa	14.79 ± 0.73Aa	0.47 ± 0.05Aa
		P_m_	28.87 ± 1.90Bb	25.85 ± 2.85Bb	12.27 ± 3.05Ba	0.32 ± 0.11Aa
		P_h_	35.12 ± 0.20Aa	26.88 ± 0.33Bb	12.77 ± 1.07Ba	0.34 ± 0.03Aa
	Si_h_	P_0_	47.82 ± 1.18Aa	32.80 ± 1.20Aa	14.53 ± 4.69Aa	0.48 ± 0.17Aa
		P_m_	37.13 ± 1.53Ab	35.33 ± 6.33Aa	15.48 ± 2.11ABa	0.56 ± 0.17Aa
		P_h_	30.93 ± 0.66Bb	29.79 ± 0.10Aa	14.06 ± 1.93Ba	0.42 ± 0.06Aa
		**Mean ± s.d**.	**31.76 ± 8.20AB**	**27.54 ± 5.41AB**	**13.67 ± 1.72A**	**0.37 ± 0.06B**
**Stem**	Si_0_	P_0_	5.86 ± 1.45Ba	4.73 ± 0.67Ba	14.34 ± 2.53Aa	0.07 ± 0.02Ba
		P_m_	7.07 ± 2.51Aa	5.59 ± 1.34Ba	17.59 ± 0.70Aa	0.10 ± 0.02Aa
		P_h_	8.60 ± 3.73Aa	7.00 ± 3.11Ca	17.69 ± 4.49Aa	0.13 ± 0.09Aa
	Si_m_	P_0_	11.85 ± 1.94Aa	10.41 ± 0.78Aa	13.50 ± 2.98Ab	0.14 ± 0.04Aa
		P_m_	6.58 ± 1.61Ab	5.56 ± 1.84Bb	17.60 ± 0.47Aa	0.10 ± 0.03Aab
		P_h_	7.87 ± 0.51Ab	5.55 ± 0.40Bb	11.91 ± 1.60Ab	0.07 ± 0.01Ab
	Si_h_	P_0_	11.06 ± 1.75Aa	10.17 ± 0.85Aa	12.96 ± 1.35Aa	0.13 ± 0.02Aa
		P_m_	9.83 ± 2.74Aa	8.79 ± 0.73Aa	14.80 ± 4.79Aa	0.13 ± 0.05Aa
		P_h_	9.05 ± 1.48Aa	6.20 ± 0.68Ab	11.76 ± 1.24Aa	0.07 ± 0.02Aa
		**Mean ± s.d**	**8.64 ± 2.02C**	**27.54 ± 5.41AB**	**13.67 ± 1.72A**	**0.37 ± 0.06B**
**Flag leaf**	Si_0_	P_0_	23.63 ± 1.86Cb	19.04 ± 2.35Ca	14.13 ± 0.68Aa	0.27 ± 0.05Ba
		P_m_	25.09 ± 3.40Ca	19.72 ± 1.44Ba	15.57 ± 4.25Aa	0.31 ± 0.11Aa
		P_h_	24.02 ± 2.28Ca	19.59 ± 3.18Aa	16.04 ± 0.33Aa	0.31 ± 0.06Aa
	Si_m_	P_0_	35.21 ± 0.33Ba	32.00 ± 0.69Ba	12.02 ± 1.30Ab	0.39 ± 0.05ABa
		P_m_	26.02 ± 3.66Bb	28.53 ± 4.15Aa	11.38 ± 0.74Ab	0.33 ± 0.07Aa
		P_h_	37.20 ± 4.46Aa	27.48 ± 6.25Aa	15.23 ± 1.14Aa	0.42 ± 0.13Aa
	Si_h_	P_0_	48.16 ± 6.87Aa	42.08 ± 4.89Aa	13.34 ± 3.14Aa	0.57 ± 0.20Aa
		P_m_	35.88 ± 5.59Ab	32.00 ± 5.26Ab	11.74 ± 0.17Aa	0.38 ± 0.07Aa
		P_h_	31.14 ± 1.48Bc	26.33 ± 0.75Ab	13.54 ± 4.26Aa	0.36 ± 0.12Aa
		**Mean ± s.d**.	**31.82 ± 8.15AB**	**27.48 ± 7.45AB**	**15.21 ± 2.11A**	**0.41 ± 0.09AB**

Different lowercase letters indicate significant differences among the treatments in different P treatments and rice plant parts at a given Si level, respectively [least significant difference (LSD) test; p < 0.05, n = 5]. Different uppercase letters indicate significant differences among the treatments in different Si treatments and rice plant parts at a given P level, respectively (LSD test; p < 0.05, n = 5). Uppercase letters of bolded texts indicate significant differences among different plant parts (leaf, flag leaf, sheath, and stem).

**Table 5:** Two-way analysis of variance (ANOVA) of silicon-phosphorus (Si-P) levels on the contents of SiO_2_, phytolith, organic carbon (OC) associated with phytolith as expressed per unit mass of phytolith (OCphyt) and of plant dry matter (OCpdm), as well as the stock of phytolith and OC_pdm_ in different plant parts (leaf, flag leaf, sheath, and stem).

Parameters	Main factor	Leaf		Flag leaf		Stem		Sheath	
		*F*	*p*	*F*	*p*	*F*	*p*	*F*	*p*
SiO^2^ content (mg g^–1^)	Si fertilization	64.341	0.000	30.634	0.000	3.840	0.041	390.371	0.000
	P fertilization	4.342	0.029	7.191	0.005	1.537	0.242	56.941	0.000
	Si × P fertilization	4.825	0.008	7.798	0.001	2.618	0.070	68.887	0.000
Phytolith content (mg g^–1^)	Si fertilization	66.384	0.000	36.186	0.000	7.773	0.004	33.205	0.000
	P fertilization	7.929	0.003	0.578	0.571	6.197	0.009	7.123	0.005
	Si× P fertilization	3.534	0.027	4.059	0.016	6.938	0.001	3.829	0.020
OC_phyt_ (mg g^–1^)	Si fertilization	1.781	0.197	6.544	0.007	3.643	0.047	3.025	0.074
	P fertilization	1.375	0.278	0.044	0.957	3.674	0.046	1.984	0.167
	Si × P fertilization	0.883	0.494	0.619	0.655	1.194	0.347	0.681	0.614
OC_pdm_ (mg g^–1^)	Si fertilization	6.257	0.009	2.942	0.078	0.234	0.794	3.889	0.039
	P fertilization	3.606	0.048	0.245	0.785	0.809	0.461	1.054	0.369
	Si × P fertilization	1.736	0.186	1.775	0.178	2.749	0.060	1.797	0.173
Phytolith stock (mg pot^–1^)	Si fertilization	13.068	0.002	14.071	0.002	2.880	0.108	12.156	0.003
	P fertilization	0.796	0.481	0.826	0.469	0.910	0.437	2.016	0.189
	Si × P fertilization	0.608	0.667	1.664	0.241	1.893	0.196	0.859	0.524
OC_pdm_ stock (mg pot^–1^)	Si fertilization	22.866	0.000	25.016	0.000	1.850	0.186	14.224	0.000
	P fertilization	4.383	0.028	3.851	0.041	4.015	0.036	9.072	0.002
	Si × P fertilization	5.426	0.005	7.127	0.001	8.494	0.000	3.158	0.039

**Table 6:** Two-way analysis of variance (ANOVA) of silicon-phosphorus (Si-P) levels on the rice shoot biomass, stock of phytolith, and OC_pdm_ in rice shoot.

Total amount (mg pot^–1^)	Main factor	*F*	*p*
Biomass	Si fertilization	0.192	0.827
	P fertilization	2.774	0.089
	Si × P fertilization	0.270	0.894
Phytolith	Si fertilization	30.343	0.000
	P fertilization	1.920	0.202
	Si × P fertilization	2.440	0.123
OC_pdm_	Si fertilization	41.540	0.000
	P fertilization	3.322	0.059
	Si × P fertilization	14.340	0.000

### Organic Carbon Content Associated With Phytoliths Formed in Rice Plants

**Figure 3:**
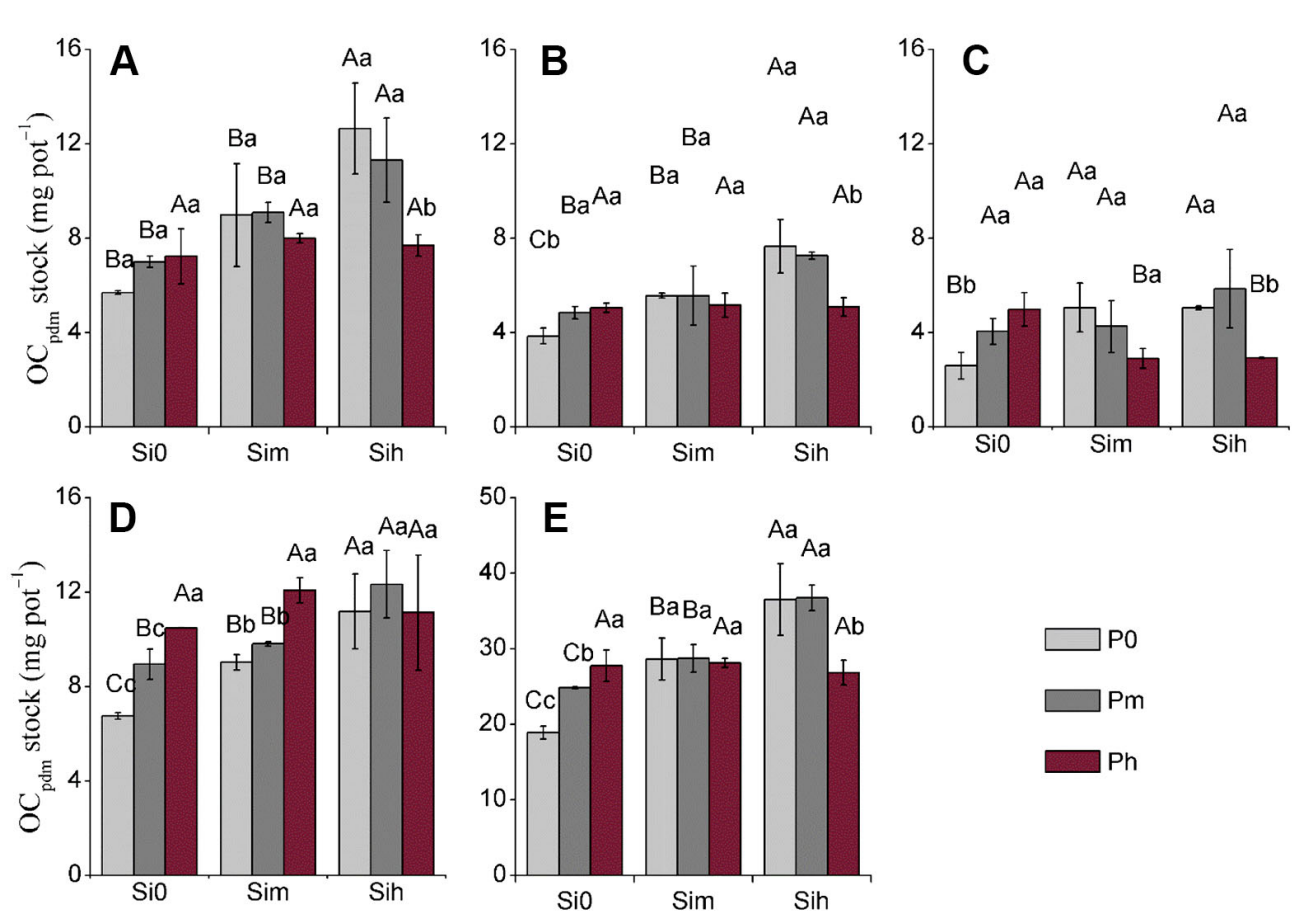
Stock of OC_pdm_ (mg pot^−1^) at two-way analysis of variance of silicon-phosphorus (Si-P) levels in the different plant parts. **(A)** Leaf; **(B)** flag leaf; **(C)**: stem; **(D)**: sheath; **(E)** rich shoot. Error bars represent the standard deviations of the means. Different lowercase letters indicate significant differences among the treatments in different P treatments and rice plant parts at a given Si level, respectively [least significant difference (LSD) test; p < 0.05]. Different uppercase letters indicate significant differences among the treatments in different Si treatments and rice plant parts at a given P level, respectively (LSD test; *p* < 0.05, n = 5).

Considering all plant parts, OC_phyt_ ranged from 11.16 to 18.17 mg g^–1^, but did not differ between Si-P treatments and plant parts (**Tables 4**–**6**). OC_phyt_ content did not vary following P application irrespective of the Si supply (Si_0_, Si_m_, and Si_h_), while this effect was not true for stem and flag leaves with a significant increase from Si_m_P_0_ to Si_m_P_m_ and Si_m_P_0_ to Si_m_P_h_, respectively (**Table 5**). At a given level Si_h_ in leaf, and a given level Si_m_ in stem, the increase in P application rate significantly decreased their OC_pdm_ content (**Table 5**). At a given level Si_0_, the increase in P application rate significantly increased the OC_pdm_ stock in all plant parts as well as plant shoot biomass except leaves, while at a given level Si_h_ level, the increase in P application rate significantly decreased the OC_pdm_ stock in all plant parts as well as plant shoot biomass except sheath (**Table 6** and **Figure 3**). However, OC_pdm_ content and its stock significantly increased with increasing Si application rate due to the increased phytolith content and phytolith stock in all plant parts, respectively (**Table 4** and **Figure 3**).

## Discussion

### Effects of Silicon-Phosphorus Supply on Rice Shoot Biomass and Yield

Our experimental data show that the addition of P alone increased biomass and grain yield (a significant increase from Si_0_P_0_ Si_0_P_m_ Si_0_P_h_); but when a combined Si-P fertilization were applied there was no significant increase in biomass and yield except that at Si_m_P_m_ and Si_m_P_h_ (**Table 3**). This supports the results of previous experiments carried out either in the field (Liu et al., 2014; Liang et al., 2015; Song et al., 2015) or in pots (Agostinho et al., 2017; Liang et al., 1994; Ma and Takahashi, 1990). Si fertilizer supply increased the stock of bioavailable Si that is crucial for sustainable paddy rice yield production (Klotzbücher et al., 2015). Furthermore, once available Si is taken up by plant roots, the accumulation of phytoliths in plant tissues can enhance the efficiency of plant photosynthesis and water use (Meunier et al., 2017), as well as their tolerance to biotic stresses (Epstein, 1994; Cooke and Leishman, 2016; Coskun et al., 2019). On the other hand, P supply likely increased plant growth and fecundity as well as root growth (Lambers et al., 2006; Brown et al., 2012). Indeed, low P levels (i.e., Si_m_P_0_ or Si_h_P_0_; **Table 3**) did not significantly increase rice biomass regardless of plant part (**Tables 2** and **3**), confirming that rice growth was clearly limited at low P supply (Ma and Takahashi, 2002; Ma, 2004; Cooke and Leishman, 2016; Agostinho et al., 2017; Hu et al., 2018) even with increasing the addition of Si fertilizer. Excessive inorganic P within rice plant inhibits enzyme reactions, induces abnormal osmotic pressure in plant cell, which further decreases rice growth (Ma and Takahashi, 1990). As reported by Ma and Takahashi (1990), the levels of bioavailable P and Si in soil influence plant P content. At Si_0_ level, the increase in P supply did not result in a change of stem, sheath and flag leaf biomass (**Table 3**) likely because the positive side-effects of P nutrition were limited at a high P supply, as mentioned here above. However, these side-effects may have been enhanced by low Si level. Yet once available P content increases up to 17.8–20.3 mg kg^−1^ at P_h_ level (**Table 2**), the increase in bioavailable Si is beneficial to rice plants by decreasing P uptake (data not shown; Ma and Takahashi, 1989; Owino-Gerroh and Gascho, 2005; Greger et al., 2018), which, in turn, decreases plant P content (Ma and Takahashi, 1990). This Si-induced decrease in plant P uptake can also result from the molecular mechanism of down-regulating the expression of P transporter gene, *OsPT6* in rice (Hu et al., 2018). The Si-P interaction thus contributes to increase rice biomass at Si_m_P_m_, Si_m_P_h_, and Si_h_P_h_ levels (**Table 5**), suggesting Si supply may alleviate excessive P application.

### Effects of Silicon-Phosphorus Supply on the Production of Phytoliths

**Figure 4:**
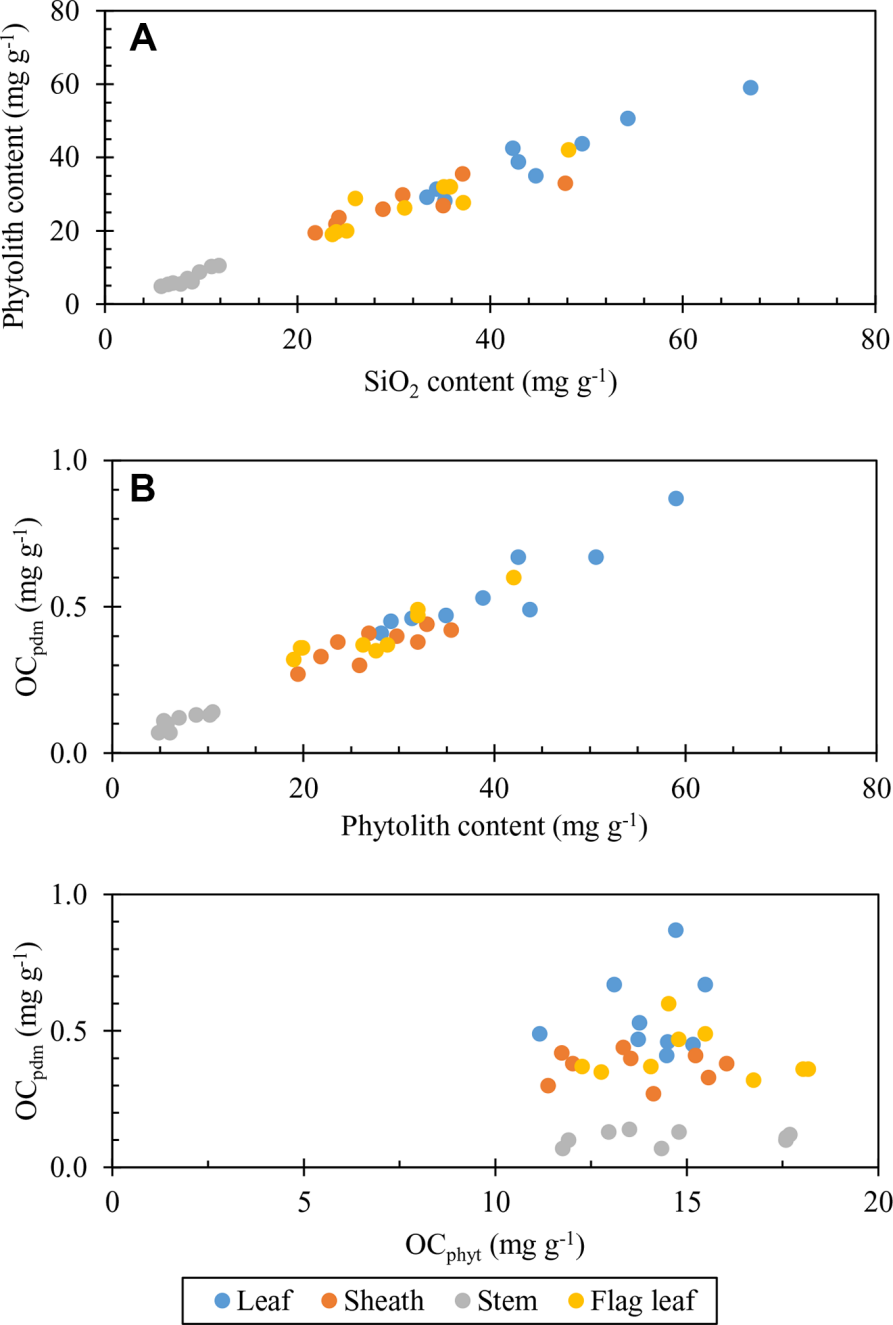
Plot of: **(A)** phytolith content of plant parts against SiO_2_ content considering all silicon-phosphorus (Si-P) treatments (leaf: y = 0.9151x−1.2668, *R*^2^ = 0.9254 P < 0.01; flag leaf: y = 0.8248x + 1.3865, *R*^2^ = 0.8035 P < 0.01; Sheath: y = 0.5457x + 10.337, *R*^2^ = 0.6938 P < 0.01; Stem: y = 1.0171x−1.6823, *R*^2^ = 0.8929 P < 0.01). **(B)** OC_pdm_ content of plant parts against phytolith content considering all Si-P treatments (leaf; y = 0.0134x + 0.0233, *R*^2^ = 0.8557 P < 0.01; flag leaf; y = 0.011x + 0.1038, *R*^2^ = 0.8097 P < 0.01; sheath; y = 0.008x + 0.1541; R^2^ = 0.6845 P < 0.01; stem; y = 0.0121x + 0.0166; *R*^2^ = 0.7924 P < 0.01). **(C)** OC_pdm_ content of plant parts against C content of phytoliths (OC_phyt_) considering all Si-P treatments (leaf; y = 0.019x + 0.29, *R*^2^ = 0.0273 P > 0.05; flag leaf; y = −0.0079x + 0.5291; *R*^2^ = 0.0329 P > 0.05; sheath; y = −0.003x + 0.4191; stem; y = 0.0027x + 0.0629; *R*^2^ = 0.0491 P > 0.05).

At a given P level, Si_2_O content significantly increased with increasing Si application rate compared to control (Si_0_), regardless of plant part. Thus, the addition of Si fertilizer as monosilicic acid (H_4_SiO_4_) taken up by roots resulted in silica accumulation in plant tissues through the formation of phytoliths (**Figure 4A**). This significant increase was due to the addition of Si fertilizer that can improve the well-observed increase in plant available Si in soils (**Table 2**). The DSi release from highly soluble Na_2_SiO_3_, wollastonite and other Si fertilizers (Haynes et al., 2013; Haynes, 2014; Keeping, 2017; Li et al., 2018b; Li et al., 2019) largely contributed to the pool of bioavailable Si, from which it was taken up by plant roots to accumulate around plant transpiration termini. As expected, P fertilizer supply did not change the concentration of available Si in Si_0_ level (**Table 2**), and thus of phytolith content, regardless of plant part (**Table 4**). Interestingly, our data further show that, at given levels Si_m_ and Si_h_, the increase in P application rate decreased the formation of phytoliths, but not always significantly, and regardless of plant part, except in flag leaf at Si_h_ treatment (**Table 4**). This trend is in accordance with Ma and Takahashi (1990) who reported that Si content of rice shoots decreased with increasing P availability in soil (**Tables 2** and **3**). As here discussed above, this trend of decreasing Si deposition in plant tissues resulted from dilution caused by increased plant growth following P application and the molecular mechanism of down-regulating the expression of P transporter gene, OsPT6 in rice (Hu et al., 2018). Since shoot biomass significantly increased following P addition, our data thus suggest that combined Si-P fertilization contributes to increased Si bioavailability in soil, Si root uptake, phytolith formation, and rice plant biomass, which, in turn, increases the stock of phytolith production in plants, while this effect is limited at the high P levels.

### Effects of Silicon-Phosphorus Fertilization on Carbon Associated With Rice Phytoliths

Considering all plant parts (**Figure 4**), our data suggest that OC_pdm_ may be controlled by phytolith accumulation in plant tissues (**Figures 4A, B**), during which the incorporation of OC seems to be constant (**Figure 4C**) and therefore does not influence the OC content of phytoliths, OC_phyt_, in line with previous hypotheses (Li et al, 2013c). Evidently, the increase in phytolith stock increases the stock of OC_pdm_, i.e., the quantity of OC associated with phytolith in living plant tissues.

Si-P fertilization does not affect OC_phyt_ content, regardless of plant part and biomass whereas it affects OC_pdm_ (**Table 4**). SEM-energy dispersive X-ray spectroscopy (EDX) image (**Figure 1**) illustrates that OC can be associated with the extracted phytoliths. However, the associated OC levels, irrespective of its source, do not change with the fertilizer treatments. SEM-EDX is semi quantitative, and thus, we used this technique not to quantify but to check the OC content as determined chemically. Therefore, we may not conclude about the possible entrapment of OC during polymerization of biogenic amorphous silica as previously proposed (Hodson et al., 1985; Parr and Sullivan, 2005; Zuo and Lü, 2011; Parr and Sullivan, 2014; Alexandre et al., 2015; Alexandre et al., 2016; Reyerson et al., 2016; Hodson, 2016; Song et al., 2016). Similarly, the hypothetical ability of plant phytoliths to occlude OC does not vary depending on the application rate (this study) and type of Si supply: basalt powder (Guo et al., 2015) or slag-based silicate (Song et al., 2015). According to Zhao et al. (2016), increased N supply in degraded grasslands decreased the phytolith content in grass shoots, while significantly increased OC content of their phytoliths. These authors hypothesized that the increase in OC_phyt_ was probably caused by improved cell growth, partly enlarged cell volume and decrease in the specific surface area of phytoliths. Similarly, Gallagher et al. (2015) reported, that growing conditions impact the OC content of phytoliths in *Sorghum bicolor* irrespective of the type and rate of application of inorganic fertilizers. These growth conditions, referring to different nutritive regimes of N, P, K, and microelements, affected the plant transpiration stream, and thus Si accumulation (Gallagher et al., 2015), which in turn, affect the OC content of phytolith (Blackman, 1969; Hodson et al., 1985). In addition to the growth conditions, the nature of plant part or organ might influence the phytolithic OC content through its impact on phytolith morphology and specific surface area (Li et al., 2013c and Li et al., 2014; **Table 4**).

Although Si-P fertilization did not increase OC_phyt_, the application of Si and P fertilizer can substantially improve the OC_pdm_ content in rice plant through increasing phytolith accumulation (**Figures 2** and **3**; p < 0.001). Our data further show that the content of phytolithic OC in rice plants mainly depends on Si supply. Indeed, phytolith accumulation in rice plant tissues significantly increased with increasing supply of Si fertilizer. Thus, regulating Si supply promoted the OC content associated within phytolith by increasing phytolith accumulation in plant notably through the increase in biomass production. Consequently, increasing crop productivity could play a crucial role in increasing the stock of phytolithic OC, while the processes explaining OC associated within phytoliths are still debated. Here the largest rice biomass was obtained at Si_h_P_m_ level (Si = 0.52 g kg^–1^; P = 0.2 g kg^–1^), regardless of plant part (**Table 3**). The level Si_h_P_m_ largely contributed to double the stock of phytolithic OC (mg pot^−1^) from 18.9 at Si_0_P_0_ to 36.8 at Si_h_P_m_ (**Figure 3E**). Another lesson is that P should not be neglected if rice productivity is to be improved as discussed above. Thus, regulating Si-nutrient supply combined with optimal P supply is promising to enhance both phytolith formation and associated organic carbon in Si-accumulating plants, as well as crop productivity.

## Conclusion

Our experimental results show that i) phytolith concentration increases with increasing Si fertilization, ii) phytolithic OC concentration does not depend on Si or P fertilization, iii) as the biomass increases with Si fertilization, the stocks of phytolith and phytolithic OC increase, iv) P fertilization has no clear impact either on phytolith or phytolithic OC concentration, but increases plant biomass and grain yield. Despite the occurrence of OC associated within phytoliths, we cannot be sure of OC occlusion within phytoliths. We conclude that the combined Si-P fertilization increases the phytolith stock by increasing the biomass and phytolith content of rice plants. Through these positive effects, combined Si-P fertilization may thus address agronomic (e.g., sustainable ecosystem development) and environmental (e.g., climate change) issues through the increase in crop yield and phytolith production as well as the promotion of Si ecological services and OC accumulation within phytoliths.

## Author Contributions

We thank Mrs. Linan Liu and Mr. Xiaomin Yang for laboratory assistance (Tianjin University). ZL and FG carried out the experiment, analyzed all data and prepared the draft. XW and ZS guided the experiment and revised manuscript. J-TC and BD reworked and revised the manuscript. All authors played a significant role in the development of the study and in writing of the manuscript. The submitted version of the manuscript has been read and accepted by all co-authors.

## Funding

The work was supported by National Natural Science Foundation of China (41571130042, 41930862 and 41522207) and the State's Key Project of Research and Development Plan of China (2016YFA0601002, 2017YFC0212703). ZL is supported by ASP (aspirant)-FNRS of Belgium in 2015–2019 and was also supported by Fonds spécial de recherché of UCLouvain (UCLouvain-FSR) in 2014–2015. The authors declare no competing financial interests.

## Conflict of Interest

The authors declare that the research was conducted in the absence of any commercial or financial relationships that could be construed as a potential conflict of interest.
